# Family Health Clinical Officers: Key professionals to strengthen primary healthcare in Kenya

**DOI:** 10.4102/phcfm.v16i1.4594

**Published:** 2024-07-29

**Authors:** Katherine Linley

**Affiliations:** 1 School of Clinical Medicine and Surgery, Kijabe College of Health Sciences, Division of Education and Research, AIC Kijabe Hospital, Kijabe, Kenya

**Keywords:** clinical officer, primary healthcare, family health, higher diploma, AIC Kijabe, Kenya

## Abstract

Primary healthcare (PHC) is recognised as the means to achieve universal health coverage, a national priority for Kenya. With only approximately 200 family physicians for a population of over 54 million, innovative solutions for providing quality PHC are needed. Clinical Officers, as mid-level health workers, already provide much of the primary care across Kenya, but without specialised training. To provide highly trained Family Health practitioners, a Higher Diploma in Family Health for Clinical Officers (FHCO) was launched by the government in 2018. With experience in delivering innovative and strategic higher diplomas, AIC Kijabe Hospital has been involved in curriculum development of this new diploma since its inception, and in October 2021 the first cohort of FHCO trainees was admitted to Kijabe College of Health Science, graduating in 2023. The second cohort is underway with plans for an annual intake. The FHCO graduates are running Family Medicine clinics at AIC Kijabe Hospital and its satellite clinics and are heavily involved in teaching. They are well-trained to deliver comprehensive, evidence-based, cost-effective and holistic care. As the programme expands, we expect graduates to be working across the country and leading efforts in enhancing the health and well-being of individuals, families and communities within primary healthcare networks (PCNs). By training FHCOs, this higher diploma is an efficient and cost-effective way to improve PHC, particularly for underserved Kenyans, and thus is a key part of enabling the Kenyan Government to achieve universal health coverage. This model of training could easily be replicated in other countries.

## Introduction

To achieve universal health coverage (UHC), as per Sustainable Development Goal 3 (target 3.8), the World Health Organization (WHO) recommends the reorientation of health systems towards primary healthcare (PHC).^1^ It is well established that PHC-oriented health systems produce better outcomes, enhanced equity and improved efficiency. As the WHO explains, ‘Scaling up primary health care interventions across low- and middle-income countries could save 60 million lives and increase average life expectancy by 3.7 years by 2030’.^2^

## Primary healthcare in Kenya

Kenya continues to face tremendous challenges to the health of its people despite sustained efforts through government, church, non-governmental organisation and private sector. The Kenyan government has made UHC a priority, focusing on decentralising healthcare to the community level and on prevention and promotion in tackling infectious diseases, rising rates of non-communicable diseases and high maternal and child mortality.^3^ This will be especially important in addressing the large inequities of access for the majority of the population that live in rural areas and who are often forced to travel long distances in search of quality healthcare.

The Ministry of Health, through the Kenya PHC Strategic Framework 2019–2024, outlines PCNs as the best model for the provision of PHC service at the community level, based on the Cuban model.^4^ To strengthen the PHC system in this way, qualified, skilled and equitably distributed health workers are vital and investment is key.^5^

Family physicians are an important part of the PCN model, but there are only approximately 200 family physicians in Kenya for a population of over 54 million. However, for several decades, clinical officers (COs), who are non-physician clinicians, or mid-level health workers, have been a key part of the health service in Kenya.^6^ Clinical Officers currently provide much of the primary care across Kenya but have only a short time covering PHC during their basic training. In 2020, Kenya had 25 000 COs and 13 000 doctors and a higher percentage of COs work in rural areas compared to doctors.^4,6^ This is consistent with many other countries in sub-Saharan Africa, where health systems are often reliant on mid-level health workers, such as COs.^7^ Also, mid-level health workers are more likely than doctors to stay in underserved areas and require shorter training courses, making investment in their training worthwhile.^8^ Interestingly, a systematic review found no difference in quality of care provided between mid-level and higher-level health workers.^9^

Clinical officers have therefore been identified as key professionals who could play a major role in PCNs across Kenya.^10^ It is recognised that curriculum reform for mid-level health worker programmes in Africa is needed; with updates to both content and pedological approach required.^11^ So, the idea of a higher diploma in Family Health for Clinical Officers (FHCO) was developed and launched by the government in 2018 at Kenya Medical Training College. With such a small number of family physicians in Kenya, it makes sense to utilise clinical officers in this role.

## Delivering training in Family Health for Clinical Officers at AIC KIjabe Hospital

In recent years, AIC Kijabe Hospital has pioneered other innovative higher diplomas for COs such as the higher diploma in Emergency and Critical Care. Recognising the need for a similar higher diploma in Family Health, family physicians at AIC Kijabe Hospital were involved with curriculum development for the FHCO higher diploma from the start. In October 2021, the first cohort of FHCO trainees was admitted to Kijabe College of Health Science, graduating in 2023. The second cohort is currently underway with plans for an annual intake.

The FHCO higher diploma is an 18-month programme with a vision to create clinicians who can take care of individuals, families and communities in a wide range of pathologies and to differentiate those who require urgent intervention or referral. The aim is that graduates will be experts in the field of family health. We focus on clinical skills such as communication, physical examination and decision-making. There is an emphasis on patient-centred care, evidence-based medicine and lifelong learning. Classroom teaching occurs during week-long blocks using multimedia with pre-reading and modular exams. Leveraging on the previous clinical experience of these adult learners, we use a problem-based teaching approach to encourage participatory learning. Clinical mentorship and assessment are also undertaken during clinical placements, which are all in primary care settings, in a variety of geographical locations and levels of healthcare. There is an emphasis on community-oriented primary care. The curriculum also includes health system management, leadership, teaching skills and quality improvement methods meaning that FHCOs are equipped to train and lead others, thus expanding the impact of their training.

As champions of PHC and ideally placed to capacity-build, family physicians have developed and led this training. The FHCO graduates are now part of the faculty. The cost of the training is approximately $4000 per trainee, which is much cheaper than comparable training for physicians.

## Outcomes so far

The FHCO graduates are running family medicine clinics at AIC Kijabe Hospital and its satellite clinics to provide continuity of care for patients with multimorbidities. They are also involved in teaching FHCO trainees, COs and interns. Current trainees are from diverse areas of the country and we expect to have graduates who are increasingly dispersed as they work across Kenya. We see that through the training, FHCOs can deliver comprehensive, evidence-based, cost-effective and holistic care over a wide range of patient conditions. We believe FHCOs are well equipped to lead efforts in enhancing the health and well-being of individuals, families and communities as part of community orientated primary care teams within PCNs.

## Conclusion

The FHCO higher diploma provides a much higher level of expertise in PHC compared to the standard diploma held by COs. The training provides and equips COs to be key members of the PHC team. The efficient and focused training also provides very good value for money; not only because of the reasonable costs of training but also because COs are more likely than physicians to stay working in rural areas where quality PHC is desperately needed. The FHCO programme is therefore a highly strategic way for Kenya to strengthen PHC and so to make steps towards achieving UHC. This curriculum and model of training could be shared and replicated in many parts of the world where non-physician clinicians work.
